# Curated Collection for Educators: Five Key Papers About Second Victim Syndrome

**DOI:** 10.7759/cureus.4186

**Published:** 2019-03-06

**Authors:** Jennifer Mitzman, Christian Jones, Shannon McNamara, Megan Stobart-Gallagher, Andrew King

**Affiliations:** 1 Emergency Medicine, The Ohio State University Wexner Medical Center, Columbus, USA; 2 Surgery, Johns Hopkins University School of Medicine, Baltimore, USA; 3 Emergency Medicine, New York University Langone Medical Center, New York, USA; 4 Emergency Medicine, Thomas Jefferson University Hospitals, Philadelphia, USA

**Keywords:** junior educators, wellness, curated collection, medical education, second victim syndrome, modified delphi, faculty development, resilience

## Abstract

Second victim syndrome (SVS) is defined as the psychological or emotional suffering of healthcare workers as a result of a patient adverse, or near miss, event. Initially thought to be related to medical error, we now recognize that SVS can result from a much wider range of circumstances including adverse pediatric patient events, unanticipated deaths, or patients well known to the provider. Residents are particularly susceptible to SVS yet relatively little is written about this topic targeted at their educators. Since educators are positioned to help recognize and guide learners through the experience, this paper targets that reader audience. In this article, we identify and summarize five key papers relevant to educators interested in learning more about SVS as it relates to learners.

We identified an extensive list of papers relevant to SVS via online discussions within the Academic Life in Emergency Medicine (ALiEM) Faculty Incubator. The Faculty Incubator is a digital community of practice providing professional development for educators. This list was augmented by an open call on Twitter seen by over 2000 people and yielding a list of 31 papers. We then conducted a three-round modified Delphi process within the authorship group, which included both junior and senior clinician educators, to identify the most impactful papers for educators interested in SVS.

The three-round modified Delphi process ranked all papers submitted for review and used iterative rounds to select the five highest-rated papers for inclusion in this article. The group then summarized each of the five papers with specific consideration for junior faculty educators and faculty developers with an interest in SVS in learners.

The five papers featured in this article serve as a key reading list for educators across specialties interested in SVS and our commentary provides context for medical educators using the articles.

## Introduction and background

**Figure 1:**
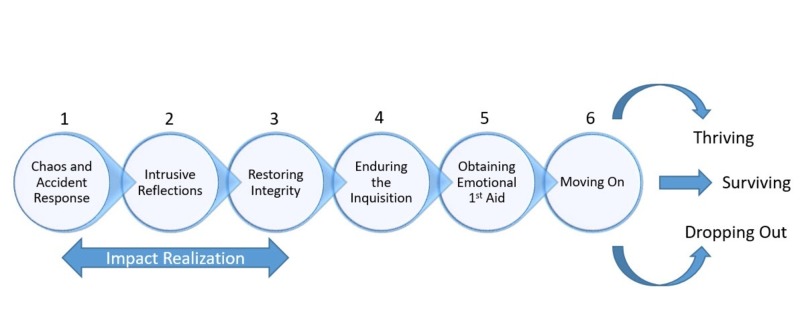
The six stages of the second victim recovery trajectory

Second victim syndrome (SVS) was coined by Dr Albert Wu in the year 2000. It is used to describe the suffering of a healthcare worker in the face of an adverse patient event, medical error or patient-related injury as a result of psychological trauma caused by the event [1-3]. While the concept of provider suffering predates Dr Wu's coining of the term, he has been a leading voice in combating the problem and his term ignited a conversation around what healthcare professionals experience. Some studies report rates as high as 76% of physicians experiencing a personal or professional impact as a result of an adverse event [3-4]. Symptoms of SVS have been described in the literature and include physical manifestations such as fatigue, disordered sleep and elevated heart rate as well as psychological and emotional distress [2,5]. These symptoms often occur most during the impact realization phase. The six stages of recovery range from initial chaos and accident response to moving on by recovery or with long-term consequences and are described in the literature (Figure 1) [2,6]. Second victim syndrome begins in stage one with the recognition of the event. People will move through predictable stages including intrusive reflection, which is often accompanied by self-doubt and guilt. In phase three, they seek acceptance and rebuild trust with their colleagues. The inquisition in stage four refers to organizational investigative processes which may cause anxiety about employment. Emotional first aid is critical to the process and ultimately the healthcare worker will move on. Their trajectory can be one of thriving despite the event, surviving but continuing to suffer as a result, or dropping out of their current environment or role [2].

Despite these clear and well-documented descriptions, recognition and institutional support lag. Burnout, depression, and suicide are associated with SVS [1,7]. Since learners are particularly susceptible, this topic is an important piece of the overall focus on wellness and growth mentality in medical education. In this article, we selected and summarized five papers that were deemed highly relevant for medical educators interested in understanding SVS.

The Faculty Incubator is a virtual community of practice, an initiative developed by Academic Life in Emergency Medicine (ALiEM) to promote professional development. Participants from this program wrote this paper to summarize the most relevant literature on the topic of SVS for both junior educators and those senior educators with a role in faculty development.

## Review

Methods

As part of the ALiEM Faculty Incubator, several scholarship groups were formed to work on projects related to medical education. We, the authors of this article, chose to focus on SVS in medical education. The group comprised three junior faculty members and two mentors with expertise and interest in SVS and medical education. The online discussions within the ALiEM virtual community of practice involved many additional junior faculty members and more experienced mentors. While the virtual discussions on SVS related to various populations occurred, one author gathered all of the literature that was exchanged and recommended within the virtual platform and compiled the articles into a list. To ensure that a broad collection of articles was compiled, we augmented the collection with an open call for additional papers using Twitter. This query supplemented papers that the experts identified as impactful via a number of individual PubMed searches. The motive of this paper was to identify the top five impactful papers on SVS for medical educators. It was not meant to provide an exhaustive list of all possible papers on the topic.

**Figure 2:**
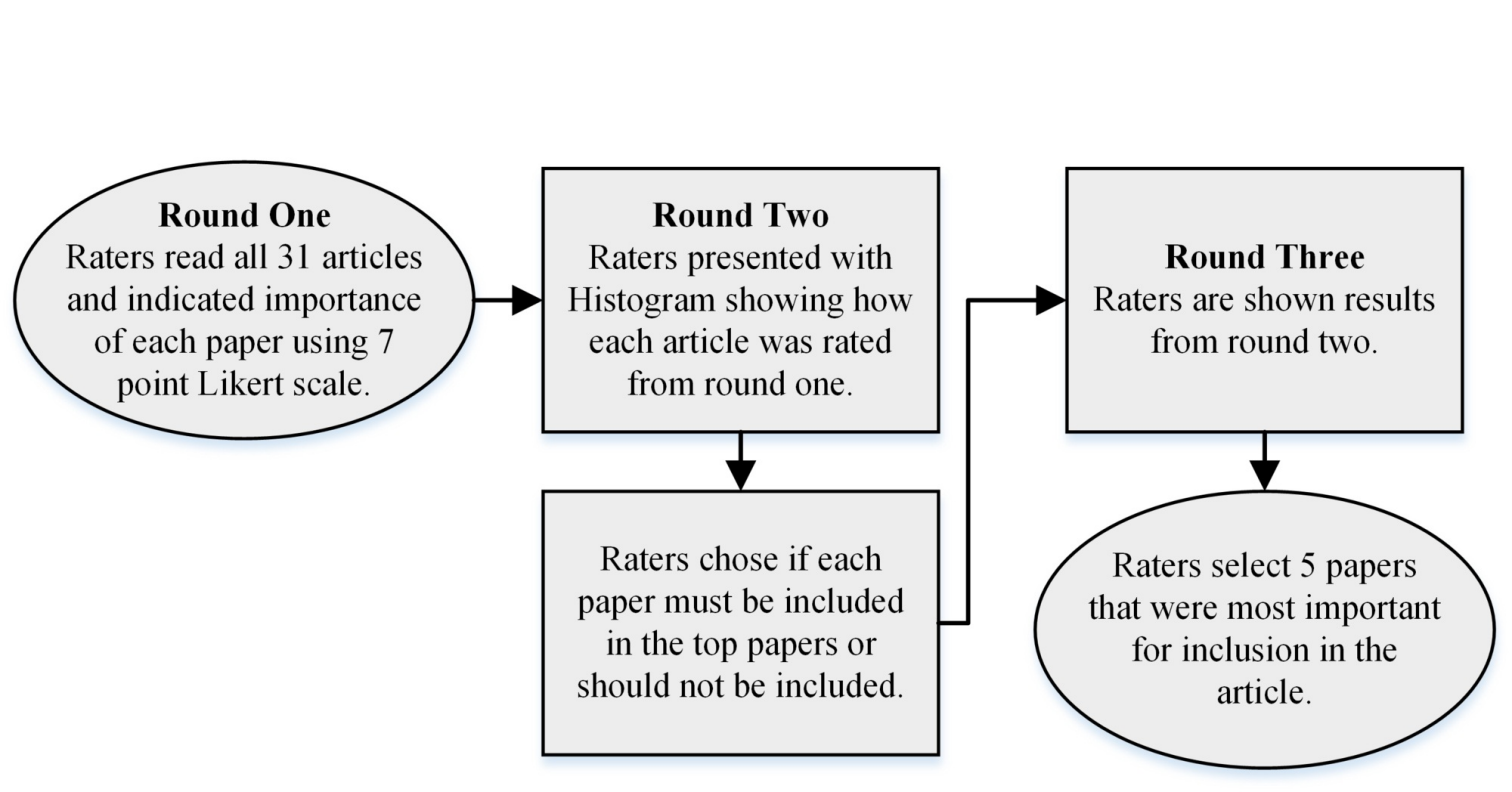
Three-round Delphi methodology

The papers on SVS were evaluated through a three-round voting process using a modified Delphi methodology, which has been previously described (Figure 2) [8]. As illustrated in Figure 1, each author read the 31 articles and actively participated in each round. In the first round, raters were instructed to indicate the importance of each article using a seven-point Likert scale. The scale was anchored at one by the statement “unimportant for or unlikely to significantly impact faculty” and at seven by the statement “essential for or illuminating and highly useful to faculty.” During the second round, raters were provided with a frequency histogram displaying how each article had been rated in the first round. Participants were then asked to dichotomously choose if each article “must be included in the top papers” or “should not be included in the top papers.” They were not limited to five for the “must include” category. In the third round, raters were provided with the results of the previous round as the percentage of raters who indicated that each article must be included. Participants were subsequently instructed to select the five papers they believed most important for inclusion in the article.

Similar methods were used by the ALiEM Faculty Incubator in a previous series of papers published in both the Western Journal of Emergency Medicine and Population Health [9-15] and Cureus [16]. This modified Delphi process does not satisfy traditional Delphi methodology as it was done virtually rather than in face-to-face sessions. Additionally, the participants included both novices (i.e., junior faculty participants in the ALiEM Faculty Incubator) and experienced medical educators (i.e., clinician educators, all of whom have published ten or more peer-reviewed publications and who serve as mentors and facilitators of the ALiEM Faculty Incubator) [8]. However, this was intentional, as we believe that the inclusion of more junior educators is essential to ensure that the selected papers would be relevant to educators at different stages of their careers.

Results

Online discussions via the ALiEM Faculty Incubator in conjunction with social media collection generated a list of 31 articles. The three-round modified Delphi process was utilized to determine the five most relevant papers. They are discussed in the discussion section of this paper. Our ratings of all 31 papers are listed in Table 1 along with their citations.

**Table 1:** The complete list of educational scholarship literature collected by the authorship team

Citation	Round 1: Initial mean scores (SD) max score 7	Round 2: % of raters who endorsed this paper	Round 3: % of raters who endorsed this paper	Top 5 papers
Marmon LM, Heiss K: Improving surgeon wellness: the second victim syndrome and quality of care. Seminars in Pediatric Surgery. 2015, 24: 315-318. https://doi.org/10.1053/j.sempedsurg.2015.08.011 [17]	6.2 (0.84)	100%	100%	1
Tamburri LM: Creating healthy work environments for second victims of adverse events. AACN. 2017, 28: 366-374. https://doi.org/10.4037/aacnacc2017996 [2]	5.6 (0.89)	100%	80%	2
Wuthnow J, Elwell S, Quillen J M, Ciancaglione N: Implementing an ED critical incident stress management team. Journal of Emergency Nursing. 2016, 42: 474-480. https://doi.org/10.1016/j.jen.2016.04.008 [18]	6.4 (0.89)	80%	80%	3
Edrees H, Connors C, Paine L, Norvell M, Taylor H, Wu AW: Implementing the RISE second victim support programme at the Johns Hopkins Hospital: a case study. BMJ open. 2016, 6: e011708. http://doi.org/10.1136/bmjopen-2016-011708 [19]	6.2 (1.79)	80%	80%	4
Pratt SD, Jachna BR: Care of the clinician after an adverse event. International Journal of Obstetric Anesthesia. 2015, 24: 54-63. https://doi.org/10.1016/j.ijoa.2014.10.001 [20]	6 (1)	80%	80%	5
Han K, Bohnen JD, Peponis T, et al.: The surgeon as the second victim? Results of the Boston intraoperative adverse events surgeons' attitude (BISA) study. Journal of the American College of Surgeons. 2017, 224: 1048-1056. https://doi.org/10.1016/j.jamcollsurg.2016.12.039 [21]	6 (1.22)	80%	20%	
Seys D, Scott S, Wu A, et al.: Supporting involved health care professionals (second victims) following an adverse health event: a literature review. International Journal of Nursing Studies. 2013, 50: 678-687. https://doi.org/10.1016/j.ijnurstu.2012.07.006 [22]	5.8 (1.64)	40%	0%	
Rodriguez J, Scott SD: When clinicians drop out and start over after adverse events. Joint Commission Journal on Quality and Patient Safety. 2018, 44: 137-145. https://doi.org/10.1016/j.jcjq.2017.08.008 [23]	5.4 (0.89)	40%	20%	
Clancy CM: Alleviating “second victim” syndrome: how we should handle patient harm. Journal of Nursing Care Quality. 2012, 27: 1-5. https://doi.org/10.1097/NCQ.0b013e3182366b53 [6]	5.4 (0.55)	80%	20%	
Wu A: The unity of medical errors. Journal of Patient Safety and Risk Management. 2018, 23(2): 49-50. https://doi.org/10.1177/2516043518765841 [24]	5.4 (1.34)	20%	0%	
Gispen F, Wu A: Psychological first aid: CPR for mental health crises in healthcare. Journal of Patient Safety and Risk Management. 2018, 23: 51-53. https://doi.org/10.1177/2516043518762826 [1]	5.2 (2.17)	40%	0%	
Hammond J, Brooks J: The world trade center attack helping the helpers: the role of critical incident stress management. Critical Care. 2001, 5: 315-317.https://doi.org/10.1186/cc1059 [5]	5 (1.22)	20%	0%	
Davidson JE, Agan DL, Chakedis S, Skrobik Y: Workplace blame and related concepts: an analysis of three case studies. Chest. 2015, 148: 543-549. https://doi.org/10.1378/chest.15-0332. [25]	5 (0.71)	60%	0%	
Elwahab SA, Doherty E: What about doctors? The impact of medical errors. The Surgeon. 2014, 12: 297-300. https://doi.org/10.1016/j.surge.2014.06.004 [26]	4.8 (1.48)	60%	0%	
Regel S: Post-trauma support in the workplace: the current status and practice of critical incident stress management (CISM) and psychological debriefing (PD) within organizations in the UK. Occupational Medicine. 2007, 57: 411-416. https://doi.org/10.1093/occmed/kqm071 [27]	4.8 (1.48)	20%	0%	
Treatment of PTSD - PTSD: National Center for PTSD (2007). Accessed 10/03/2018. www.ptsd.va.gov/professional/trauma/disaster-terrorism/debriefing-after-disasters.asp. [28]	4.6 (2.07)	40%	20%	
Pratt S, Kenney L, Scott SD, Wu AW: (2012). How to develop a second victim support program: a toolkit for health care organizations. Joint Commission Journal on Quality and Patient Safety. 2012, 38: 235-240. https://doi.org/10.1016/S1553-7250(12)38030-6 [29]	4.4 (1.95)	0%		
Coughlan B, Powell D, Higgins MF: The second victim: a review. European Journal of Obstetrics & Gynecology and Reproductive Biology. 2017, 213: 11-16.https://doi.org/10.1016/j.ejogrb.2017.04.002 [3]	4.4 (0.55)	0%		
Sending Out an SOS. (2018). Accessed 10/03/2018. www.hopkinsmedicine.org/news/publications/hopkins_medicine_magazine/features/spring-summer-2018/sending-out-an-sos [30]	4.2 (0.84)	0%		
Eklöf M, Törner M, Pousette A: Organizational and social-psychological conditions in healthcare and their importance for patient and staff safety. A critical incident study among doctors and nurses. Safety Science. 2014, 70: 211-221. https://doi.org/10.1016/j.ssci.2014.06.007 [31]	3.8 (2.17)	0%		
Greenberg N: A critical review of psychological debriefing and a proposal for the future. Journal of the Royal Naval Medical Service 2001, 87: 158-61. [32]	3.8 (0.45)	0%		
Lane MA, Newman BM, Taylor MZ, O'Neill M, Ghetti C, Woltman RM, Waterman AD: Supporting clinicians after adverse events: development of a clinician peer support program. Journal of Patient Safety. 2018, 14: e56-e60. https://doi.org/10.1097/PTS.0000000000000508 [33]	3.6 (2.19)	0%		
Quillivan RR, Burlison JD, Browne EK, Scott SD, Hoffman JM: Patient safety culture and the second victim phenomenon: connecting culture to staff distress in nurses. The Joint Commission Journal on Quality and Patient Safety. 2016, 42: 377-384. https://doi.org/10.1016/S1553-7250(16)42053-2 [34]	3.6 (1.14)	0%		
Treiber LA, Jones JH: Making an infusion error. Journal of Infusion Nursing. 2018, 41: 156-163. https://doi.org/10.1097/NAN.0000000000000273 [35]	3.4 (0.89)	0%		
Schrøder K, la Cour K, Jørgensen JS, Lamont RF, Hvidt NC: Guilt without fault: a qualitative study into the ethics of forgiveness after traumatic childbirth. Social Science & Medicine. 2017, 176: 14-20. https://doi.org/10.1016/j.socscimed.2017.01.017 [36]	3.4 (1.14)	0%		
Grissinger M. Too many abandon the “second victims” of medical errors. Pharmacy and Therapeutics. 2014, 39: 591. [7]	3.4 (1.52)	0%		
Bisson JI: Psychological debriefing for adults. Effective treatments for PTSD: Practice Guidelines from the International Society for Traumatic Stress Studies. Foa E (ed): Guilford Press, 2008. 2: 83-105. [37]	3.4 (1.34)	0%		
Chung AS, Smart J, Zdradzinski M, Roth S, Gende A, Conroy K, Battaglioli N: Educator toolkits on second victim syndrome, mindfulness and meditation, and positive psychology: The 2017 Resident Wellness Consensus Summit. Western Journal of Emergency Medicine. 2018, 19: 327-331. http://doi.org/10.5811/cpcem.2017.11.36179. [38]	3 (2.55)	0%		
Davis J: Critical incident stress debriefing from a traumatic event. Psychology Today. 2013, Accessed: 10/03/2018: https://www.psychologytoday.com/us/blog/crimes-and-misdemeanors/201302/critical-incident-stress-debriefing-traumatic-event [39]	2.8 (1.48)	0%		
Müller-Leonhardt A, Mitchell SG, Vogt J, Schürmann T: Critical Incident Stress Management (CISM) in complex systems: cultural adaptation and safety impacts in healthcare. Accident Analysis & Prevention. 2014, 68: 172-180. https://doi.org/10.1016/j.aap.2013.12.018 [40]	2.8 (2.39)	0%		
A primer on critical incident stress management (CISM). (2003). Accessed 10/03/2018: https://icisf.org/a-primer-on-critical-incident-stress-management-cism/ [41]	2.4 (0.55)	0%		

Discussion

The summaries of the five papers selected by the modified Delphi process for inclusion in this paper are presented below. They are presented in the order of highest mean percentage across rounds. Each is also accompanied by commentary on the relevance to both junior faculty educators and more senior faculty developers.

1. Marmon LM, Heiss K: Improving surgeon wellness: the second victim syndrome and quality of care. Seminars in Pediatric Surgery, (2015), 24(6), 315-318. https://doi.org/10.1053/j.sempedsurg.2015.08.011

Summary

This review is widely applicable to the entire field of medicine, not just to surgeons. Marmon and Heiss use their work to introduce the phenomenon of second victim syndrome. They further explain the incidence, risk factors, characteristics, and management of second victim syndrome. They also describe the association between SVS, burnout, and depression. A summary of Susan Scott's six stages of SVS recovery is more detailed than some of the other papers selected by our review, but uses the same six stage framework. Additionally, this article includes the concept of the “third victim”, which is the involved healthcare organization and recognizes the need for the institution to “recover” from an incident as well.

Marmon and Heiss provide a framework for management of the many stages of critical incidents, including identification of those at risk for SVS, prevention of medical errors, mitigation to limit the emotional effects on second victims, support for longer-term recovery and culture change. Like others, they recommend the development of a formal system for supporting affected providers and emphasize the need for strong leadership support of such a system. They specifically emphasize the preference for this support system to utilize trained peers rather than an external employee assistance program.

Relevance for Faculty Members

Less experienced providers are at higher risk for second victim syndrome as they feel a greater responsibility and higher risk of potential consequences. While recognition of the phenomenon and appropriate mitigation at the organizational level are imperative, recognition of one’s own need for emotional support in the setting of a critical incident is paramount. Familiarity with the stages of recovery from such an event and its long-term impact may be useful for personal experiences or to assist a colleague. *Considerations for Faculty Developers*

Faculty developers may choose to use the two frameworks provided in this article to educate other providers or to help make a case for developing a response program at the departmental or institutional level. Describing the second victim syndrome’s association with burnout and depression may encourage providers to consider all of these phenomena more seriously among themselves and their colleagues. Finally, faculty developers play a key role in the promotion of institutional culture, accepting on principle that people in the medical system are imperfect and helping to both prevent critical incidents and provide support when they occur.

2. Tamburri LM: Creating healthy work environments for second victims of adverse events. AACN Advanced Critical Care. 2017, 28(4), 366-374.

Summary

This article defines the second victim phenomenon, discusses second victim suffering and the symptoms of the second victim experience. It illustrates the six-step recovery process and defines healthy work environment standards for second victim support. Tamburri begins by presenting the incidence and nature of adverse events; in fact, she states that any nurse can become involved in an adverse patient event irrespective of their role. Second victims are defined as the healthcare providers who are involved in an unanticipated adverse patient event and become victimized in the sense that the provider is traumatized by the event. These second victims experience feelings such as guilt, shame, sadness, and grief, which can lead to profound personal and professional consequences. The author also provides a detailed table of physical, emotional, psychological, and professional symptoms that second victims can experience. The article continues by describing the six-stages of recovery for second victims. The author then provides a detailed discussion on professional resources available to second victims. Specifically, she presents the Healthy Work Environment Standards for Second Victim Support, which defined six competencies that are essential for all healthcare professionals. These include skilled communication, true collaboration, effective decision-making, appropriate staffing, meaningful reaction, and authentic leadership. Helping second victims heal and thrive after an adverse event is the responsibility of every member of the healthcare team, from bedside providers to senior leaders. This article provides a valuable framework for creating second victim support systems that can transform the work environment into a safe, supportive place.

Relevance for Faculty Members

This article provides a clear definition of the second victim phenomenon experienced by nurses. Despite its nursing audience, the author team scored this paper as highly relevant to our intended audience. The author defines the specific symptoms and the stages of recovery, which are vital for healthcare professionals to identify in colleagues and understand. Often, healthcare professionals, specifically junior faculty members, feel alone and stigmatized after an adverse event. This article clearly illustrates that they are not alone, while identifying available resources and providing a framework for supporting second victims. This paper allows junior faculty members to recognize and understand the condition both in themselves and others, while learning important resources available for support.

Considerations for Faculty Developers

Tamburri provides a detailed definition of the second victim phenomenon, while presenting common symptoms. This information is absolutely vital knowledge for faculty developers not only to educate junior faculty members on the condition but also to recognize it and provide invaluable support and mentorship for junior faculty second victims. Similarly, this article provides available resources, while highlighting competencies that healthcare professionals should master in order to support colleagues who are second victims. All should embrace these competencies, but at the very least, these should be mastered by faculty developers who can educate junior faculty and provide support to colleagues in need.

3. Wuthnow J, et al.: Implementing an ED critical incident stress management team. Journal of Emergency Nursing, 2016, 42(6), 474-480.

Summary

This paper discusses one pediatric emergency department’s approach to building a Critical Incident Stress Management team. They approach SVS from the frame of Critical Incident Stress. In this frame, an adverse event is a critical incident that causes a crisis response in people who may then experience critical incident stress. Critical incident stress management (CISM) is an established, comprehensive crisis intervention system aimed at supporting traumatized emergency personnel in a formalized way.

This group decided to start a CISM program after a particularly difficult case in which a child died. The nursing staff had difficulty coping after the event and approached the department’s leadership to request a crisis team to help staff cope after similar critical events. As a level 1 trauma center and pediatric emergency department with many high acuity pediatric cases the department decided to form a CISM Team to support the wellness of the staff and assist with coping after critical events.

The authors describe the theoretical framework of CISM and why that modality was chosen for their program. The seven core components of a CISM program are reviewed: 1. Pre-crisis preparation, 2. Demobilization/informal briefings, 3. Defusing, 4. Critical incident stress debriefing (CISD), 5. Facilitate one-on-one crisis intervention, 6. Family crisis intervention, 7. Follow-up and referral. The controversy of CISD in the literature is discussed. Some studies note the potential harms of CISD, which are often attributable to untrained staff performing CISD.

The authors include details of their program development, including team composition, role clarity, activation protocols, staff training, finances and integration with existing psychological support services. The study is limited by a lack of program evaluation, as the authors note the program is early in its development but early feedback has been positive.

Relevance for Faculty Members

This article provides a clear definition and overview of the CISM framework and how CISD is incorporated. The authors outline a reproducible format for developing an inter-professional emergency department-based CISM program. As wellness becomes a priority for emergency medicine (EM) programs, this is one model of a program that addresses workplace stress in a structured and evidence-based way. For those interested in learning more about psychological debriefing or CISD, this article shows the importance of incorporating that debriefing in a larger crisis intervention framework [42].

Considerations for Faculty Developers

This article is an important contribution from nursing leaders on an inter-professional, occupational wellness program. Wellness programs should ideally address the root causes of burnout and compassion fatigue in our workplaces. By defining critical incident stress in nonjudgmental terms and modeling self-compassion through this CISM program, the authors showed the tremendous potential of wellness taken seriously.

4. Edrees H, et al.: Implementing the RISE second victim support programme at the Johns Hopkins Hospital: a case study. BMJ Open, 2016, 6(9), e011708. https://doi.org/10.1136/bmjopen-2016-011708

Summary

The team from Johns Hopkins Hospital and its associated Armstrong Institute for Patient Safety and Quality includes Albert Wu. He first described the phenomenon of second victim syndrome among healthcare providers and has been a leading voice in combating the problem. This article is the premier description of developing one of the first institutional efforts to combat second victim syndrome and describes all aspects of the program’s formation. These include completion of a baseline provider survey, development of a network of peer responders, and a phased rollout to the entire Johns Hopkins Hospital. The Resilience in Stressful Events (RISE) team slowly grew and responded to a few calls per month, addressing issues from provider burnout to patient death. The greatest challenges met by the program appeared to be less use of the system by providers than expected, which was attributed to a lack of awareness of RISE by most hospital staff rather than a lack of interest in the program. However, peer responders (who met with both individuals and groups in their encounters) generally found that their sessions were productive and helpful to the distressed providers. Importantly, the training of peer responders included not only initial orientation and education but ongoing peer development including regular group review of RISE encounters. The authors conclude that organizations should offer programs that deliver emotional support to their employees but that a sustained and multilateral push for awareness of the program is critical to its use.

Relevance for Faculty Members

This brief introduction and methods paper describes not only the need for support of second victims but also the implementation of a directed system-wide program to help provide that support. Junior faculty can assist with team formation and use either by undergoing intensive training to become a peer responder themselves or simply spreading awareness of the program and encouraging its use when encountering difficult scenarios such as medical error. While the authors note that the formation of such a program is likely to require significant leadership buy-in, junior faculty members are valuable advocates who can help describe second victim syndrome and support the response team.

Considerations for Faculty Developers

Faculty developers may have the requisite cachet within their institution to promote the initial development of a second victim response program like RISE. If so, this blueprint for building such a program, from conception to regular retrospective review, is invaluable. Recognition of the mechanisms for responding to distressed colleagues and understanding the level of training required for responders may help prevent senior faculty from dismissing or minimizing the struggles of second victims whom they identify.

5. Pratt SD, Jachna BR: Care of the clinician after an adverse event. International Journal of Obstetric Anesthesia, 2015, 24(1), 54-63.

Summary

This review article begins by providing a heart-wrenching personal account illustrating the stages of second victim syndrome when an anesthesiologist emotionally disintegrates after a woman unexpectedly died during childbirth and then is crushed by the months of subsequent investigation. Ultimately no blame was found but the damage was done. In addition to Scott's six stages of recovery, this paper utilizes a simplified four stage progression. The stages are 1) a “kick, or visceral blow to one’s core,” 2) self doubt and failure, 3) “the fall” of life spiraling out of control, and finally ends with 4) recovery. It attempts to humanize all of the studies that describe second victim syndrome. It argues that it is normal to have a strong emotional and/or physical response to trauma, through expressions of many feelings including shame, guilt, isolation, or fear. There are varying descriptions of the stages of second victim syndrome, but all are along the same lines of initial response, impact beyond oneself, and then either recovery or potential long-term damage. 

It also provides some insight into the rights of the second victim using the TRUST acronym: treatment that is just, respect, understanding, supportive care, and transparency. Second victims deserve care beyond just kindness. While institution-specific programs are limited, there are several peer support models described that can help organizations remain accountable while providing appropriate care, treatment and emotional support to victims within the health system.

Relevance for Faculty Members

Faculty members should utilize this paper in order to see a broad, yet detailed, introduction to what second victim syndrome is, those who may be at risk and the natural progression of the experience. Both the six stage and four stage systems are discussed. This paper reinforces to faculty that being cared for by the health care system is a right. Peer support models and other mechanisms to care for the second victim are a moral necessity and should be an expected faculty resource.

Considerations for Faculty Developers

Faculty developers can use the background information to describe SVS, identify who is at risk, and learn about the progression victims go through in order to help justify the initiation of institutional support systems for clinicians. The first steps are to recognize that second victims exist in every healthcare setting, and unfortunately many more are at risk. This paper also reports on an alarming 20% who do not report full recovery. Faculty developers should focus on the fact that clinicians have the right to be supported by their organizations both physically and emotionally to help prevent a cycle of error -> burnout -> error from spiraling out of control. By recognizing second victims and providing support, institutions can help to prevent the institution from becoming a third victim and limit further damage to the second victim and potential primary victims-future patients.

Limitations

Firstly, this modified Delphi process did not incorporate a systematic literature search. We utilized field expertise and crowd-sourcing within the ALiEM online community of practice to generate the list of articles for review. We believe that this process would identify key papers on the topic of second victim syndrome while keeping the list of articles trim enough to be manageable for a modified Delphi project of this nature. Given the way the search was performed, it is possible that some relevant literature was omitted.

In a pure Delphi method, all participants are already experts in the field of interest, in this case, second victim syndrome. We utilized an approach that includes both experts and junior faculty. We felt that in doing so we were able to determine what articles would be of key relevance to all medical educators, including a junior cohort.

## Conclusions

This article identified key papers on second victim syndrome and described their relevance to faculty educators and faculty developers interested in this topic. We believe this resource will be valuable in the recognition of SVS in faculty and learners, two particularly susceptible groups. Additionally it may be useful for more senior educators with the clout and experience to shape policies and increase awareness at the departmental or institutional level.
